# Mutations in *TUBA4A* lead to human zygotic arrest and early developmental failure

**DOI:** 10.1093/lifemedi/lnad032

**Published:** 2023-08-23

**Authors:** Lin Li, Linfan Mao, Zhiguo Zhang, Yiying Yu, Miaohui Ding, Yongyan Zhi, Yunxia Cao, Beili Chen, Jie Na

**Affiliations:** Central Laboratory, Beijing Obstetrics and Gynecology Hospital, Capital Medical University, Beijing Maternal and Child Health Care Hospital, Beijing 100006, China; School of Medicine, Tsinghua University, Beijing 100084, China; Reproductive Medicine Center, Department of Obstetrics and Gynecology, the First Affiliated Hospital of Anhui Medical University, Hefei 230022, China; NHC Key Laboratory of study on abnormal gametes and reproductive tract (Anhui Medical University), Hefei 230022, China; Tsinghua-Peking Center for Life Sciences, Tsinghua University, Beijing 100084, China; Zhili College, Tsinghua University, Beijing 100084, China; School of Life Sciences, Tsinghua University, Beijing 100084, China; Reproductive Medicine Center, Department of Obstetrics and Gynecology, the First Affiliated Hospital of Anhui Medical University, Hefei 230022, China; NHC Key Laboratory of study on abnormal gametes and reproductive tract (Anhui Medical University), Hefei 230022, China; Reproductive Medicine Center, Department of Obstetrics and Gynecology, the First Affiliated Hospital of Anhui Medical University, Hefei 230022, China; NHC Key Laboratory of study on abnormal gametes and reproductive tract (Anhui Medical University), Hefei 230022, China; School of Medicine, Tsinghua University, Beijing 100084, China

**Dear** **Editor,**

Oocyte meiotic maturation, the first mitosis of a zygote, and the subsequent early embryonic development process are closely linked key events for successful human reproduction. Many maternal proteins play important roles in each stage or through all three stages. In recent years, studies have found that mutations in some genes, such as *TUBB8*, *BTG4*, *CHEK1*, *PADI6*, *NLRP2*, *NLRP5*, *REC114*, and *KHDC3L* [1, 2], can lead to oocyte maturation arrest, zygote arrest, early embryonic arrest, or the three arrested phenotype spectrum in different patients, resulting in female infertility. However, the above-mentioned genes and mutations can only explain some patients, and there are still many cases where the pathogenic genetic factors are unknown.

Twelve infertile female patients, mainly characterized by zygotic arrest, were recruited in this study and were screened for likely pathogenic mutations by whole-exome sequencing. We identified three isolated patients with heterozygous variants in the *TUBA4A* gene (Fig. 1A). The proband in Trio 1 carried a c.850G > A;p.E284K variant, which was validated by Sanger sequencing and was found to be a *de novo* variant as it was absent in the proband’s parents (Fig. 1A). The other two patients carried a c.851G > A;p.E284G and a c.850G > A;p.E284K variant, respectively, validated by Sanger sequencing (Fig. 1A).

**Figure 1.**
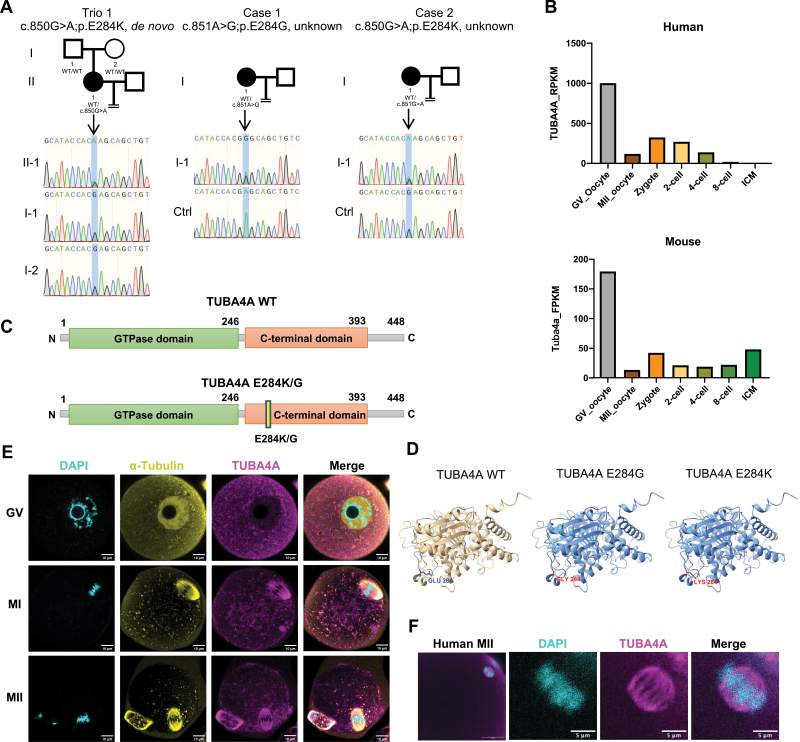
**Meiosis arrest in oocytes from TUBA4A c.850G > A and TUBA4A c.851A > G females.**(A) Left: one infertile parent-child trio with *de novo* TUBA4A. Right: two pedigrees of sporadic TUBA4A mutations. Squares indicate male family members, circles indicate female members, and black circles indicate the affected members. The “=” sign indicates infertility. (B) TUBA4A expression levels in human and mouse GV oocyte to blastocyst embryo based on RNA-sequencing data from GSE36552, and GSE71434. (C) Schematic of human TUBA4A protein domains. (D) TUBA4A protein structure illustration by AlphaFold2 and amino acid at 284 indicated by Missense 3D. WT Glutamate (GLU) is in blue. Residues Glycine (GLY) and Lysine (LYS) in E284G and E284K mutants are shown in red. (E) TUBA4A protein locates on the spindle of mouse oocytes. Cyan, DAPI; yellow, α-tubulin; magenta, TUBA4A. Scale bar, 10 μm. (F) Immunostaining of TUBA4A in human MII oocytes. Cyan, DAPI; magenta, TUBA4A. Scale bar, 5 μm.

The proband in Trio1, who was 26 years old, had undergone only one IVF cycle. 19 oocytes were retrieved, and 10 mature oocytes were fertilized with the normal sperm from her husband. All seven normal fertilized zygotes with two pronuclei (2PN) failed to progress to the cleavage stage (Table 1). Case 1 was 36 years old. She had undergone two IVF attempts and one ICSI cycle with an artificial oocyte activation (AOA) cycle. Only a few zygotes with 2PN cleaved into 2–4 blastomeres 3 days after fertilization (Table 1). She did not conceive in her embryo transfer cycle. Case 2 was 35 years old, had undergone three IVF/ICSI attempts, and obtained only a few embryos with 2–5 blastomeres (Table 1). Despite the appearance of the pronucleus after fertilization, these “normal zygotes” could not develop further.

**Table 1.** Oocyte and embryo phenotypes of the patients carrying the *TUBA4A* variant

Individual	Age (years)	Years of infertility	IVF and ICSI attempts	Retrieved oocytes	MII oocytes	0PN zygotes	2PN zygotes	Cleaved fertilized zygotes	Develop
Proband in Trio1 E284K	26	5	IVF	19	10	2	7	0	0
Case 1 E284G	36	11	IVF	16	16	3	12	4	0
			ICSI (+AOA)	3	2	0	2	0	0
			IVF	10	10	1	8	6	0
Case 2 E284K	35	14	IVF	23	23	12	8	13	0
			IVF	16	15	7	4	1	0
			ICSI	16	15	3	7	7	0

Transcriptomic analysis showed that TUBA4A is highly expressed in human and mouse oocytes (Fig. 1B). Its mRNA level decreased after fertilization, indicating it is a maternal protein. Protein sequence analysis revealed that E284 is located at the C-terminal domain, which is involved in protein binding (Fig. 1C). Structure prediction by Alphafold2 demonstrated that the conserved residue Glu-284 in the M-loops of α-tubulin (Fig. 1D), which included residues Tyr-272 to Ser-287, is involved in the lateral contact of protofilaments [3]. In the wild-type (WT) protein, the glutamic acid is a negatively charged amino acid, but the mutant lysine is a positively charged amino acid, and the other mutant glycine is a non-polar amino acid. Although they were predicted not to affect the protein’s overall structure (Fig. 1D), these mutations may disrupt the organization and dynamics of the microtubule. Immunostaining showed that TUBA4A proteins localized to the spindle and the tail of mouse and human MII oocytes and sperms, respectively (Figs. 1E-F and S1E-F). Genomic sequence analysis also showed that the TUBA4A DNA and amino acid sequences are conserved across mammalian species (Fig. S1A and S1B). According to the published translatome database [4, 5], the translation efficiency of TUBA4A in human GV oocytes is greater than 3, suggesting that it is actively translated into protein (Fig. S1C  and S1D).

To investigate how the mutant TUBA4A proteins cause female infertility, we injected mRNA encoding WT, E284G, and E284K mutants into mouse GV oocytes and let them undergo meiotic maturation (Fig. 2A). The amino acid sequence of mouse and human TUBA4A proteins are identical. Immunostaining showed that upon overexpression of WT or mutant TUBA4A, E284G oocytes had highly disrupted spindle morphology, while E284G and E284K oocytes displayed apparent chromosome misalignment (Fig. 2B). About 60% of uninjected control and WT injected oocytes reached MII, while only about 30% E284G- and E284K-injected oocytes completed meiosis I (Figs. 2C and S2A). We also investigate how TUBA4A mutants affect zygote division by mRNA microinjection. Zygotes expressing WT or mutant TUBA4A reached the two-cell stage at similar proportions (Fig. 2E) but differed in the proportion that developed to the blastocyst stage. Eighty percent of WT versus more than 60% of mutant TUBA4A embryos became blastocysts (Fig. 2F). Based on our results, we reasoned that since the mutant TUBA4A protein localized to the spindle and disrupted oocyte and preimplantation embryo division, providing more WT TUBA4A protein might outcompete the mutant protein and rescue the phenotype. Therefore, we co-injected WT and mutant mRNA mixed in a 2:1 ratio. As expected, with more WT transcripts, E284G and E284K oocytes were able to protrude the first PB at a comparable rate (approximately 60%) as uninjected control oocytes (Figs. 2G and S2B). We also tempted rescue experiments in mouse zygotes. Co-injection of WT with E284G and E284K mRNA led to a small but insignificant increase in blastocyst formation compared to E284G and E284K overexpression alone (Fig. 2H).

**Figure 2.**
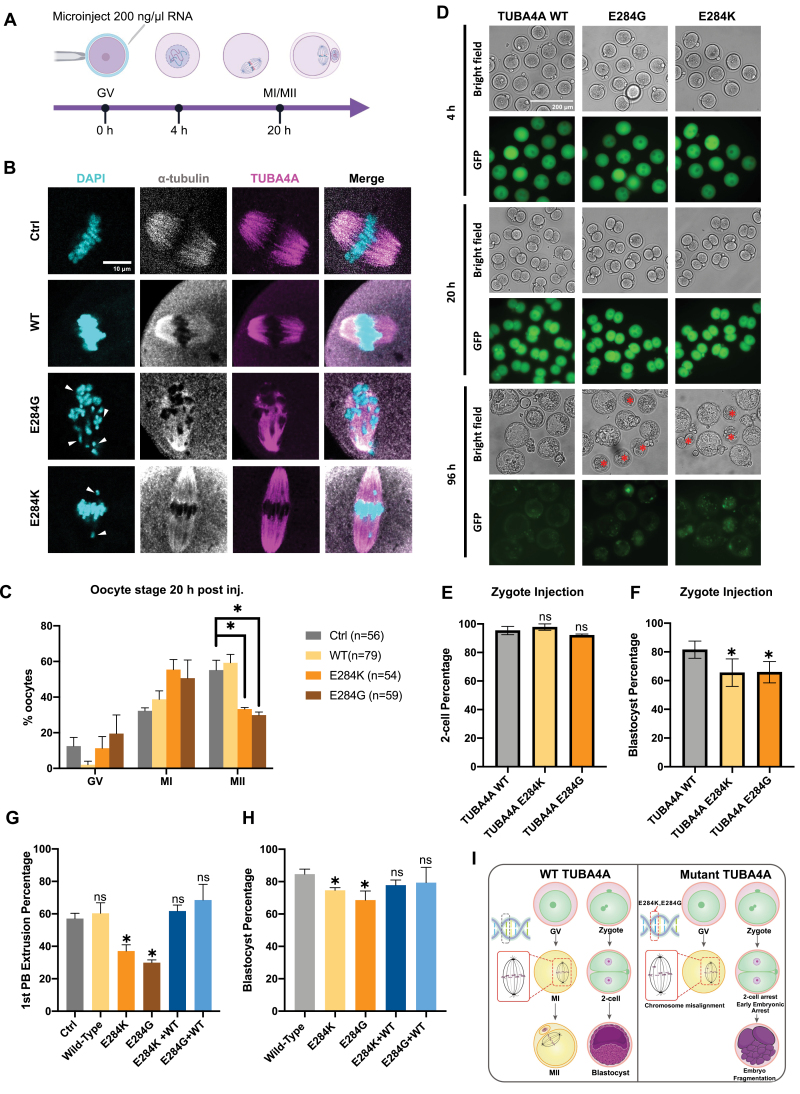
**TUBA4A E284K and E284G mutations induced spindle defects and early embryo development arrest.**(A) Schematic of expressing TUBA4A WT and E284G and E284K in mouse oocytes by mRNA microinjection (Created with BioRender.com). (B) Representative spindle immunostaining images of Ctrl, TUBA4A WT, E284G, and E284K oocytes. Cyan, DAPI; gray, α-tubulin; magenta, TUBA4A. Scale bar, 5 μm. Misaligned chromosomes were marked with white arrows. (C) Bar plot showing the percentages of GV, MI, and MII oocytes with microinjected TUBA4A WT, E284K, and E284G mRNA after 20 h in culture. Ctrl, *n* = 56. WT, *n* = 79. E284K, *n* = 54. E284G, *n* = 59. All pooled from 3 separate experiments; one-way analysis of variance (ANOVA) and Bonferroni’s test for individual comparisons. Bars are means ± standard error of the mean (SEM). GV, germinal vesicle. MI, meiosis I. MII, meiosis II. (D) Representative live cell images of zygotes microinjected with 200 ng/μL mRNA encoding TUBA4A WT, E284G, and E284K GFP fusion protein. Time is indicated. Scale bar, 100 μm. fragmented embryos were marked with a red *. (E) Bar plot showing the 2-cell ratio of zygotes injected with TUBA4A WT, E284K, and E284G mRNA. WT, *n* = 56; E284K, *n* = 66; E284G, *n* = 57. All pooled from three separate experiments; one-way ANOVA and Bonferroni’s test for individual comparisons. Bars are means ± SEM. (F) Bar plot showing the blastocyst ratio of zygotes injected with TUBA4A WT, E284K, and E284G mRNA. WT, *n* = 56; E284K, *n* = 66; E284G, *n* = 57. All pooled from three separate experiments; one-way ANOVA and Bonferroni’s test for individual comparisons. Bars are means ± SEM. (G) Bar plot showing the polar-body extrusion rates in mouse oocytes microinjected with TUBA4A WT and E284K and E284G and WT/E284K (2:1) and WT/E284G (2:1) mRNA compared with rates in a non-injected control. Ctrl, *n* = 56. WT, *n* = 79. E284K, *n* = 54. E284G, *n* = 59. WT/E284K (2:1), *n* = 45. WT/E284G (2:1), *n* = 44. All pooled from three separate experiments; one-way ANOVA and Bonferroni’s test for individual comparisons. Bars are means ± SEM. (H) Bar plot showing the blastocyst percentage of zygotes injected with E284K and E284G and WT/E284K (1:1) and WT/E284G (1:1) mRNA compared with the percentage of zygotes injected with wild-type TUBA4A. WT, *n* = 102. E284K, *n* = 65. E284G, *n* = 60. WT/E284K (1:1), *n* = 70. WT/E284G (1:1), *n* = 76. All pooled from three separate experiments; one-way ANOVA and Bonferroni’s test for individual comparisons. Bars are means ± SEM. (I) Schematic models for the effects of TUBA4A mutations on oocyte maturation and zygote development. Schematic models showed that mutant TUBA4A caused meiotic arrest in oocytes and reduced the developmental potential of zygotes.

Microtubes are composed of α- and β-tubulin, and TUBA4A is one of the many that encode α-tubulin. Previous studies have found that mutations in the protein TUBB8, which encodes β-tubulin, can lead to MI arrest in human oocytes [6]. In this study, we found that the mutation of *TUBA4A* can cause zygotic arrest and subsequent early embryonic arrest. Thus, both α- and β-tubulin play critical roles in oocyte maturation, zygote division, and early embryo development. Correct spindle assembly is essential for successful mitosis and meiosis. Only the proper assembly of the spindle can ensure the faithful separation of chromosomes and the normal development of subsequent cells and embryos. Unlike human and mouse somatic cells, there is no centrosome in human and mouse oocytes [7]. The spindle formation of mouse oocytes is mediated by acentriolar microtubule organizing centers (aMTOCs). In contrast, human oocytes have a distinctive microtubule organizing center called the human oocyte microtubule organizing center (huoMTOC) [7]. Our study found that oocytes injected with E284G mRNA exhibited abnormal spindle morphology, suggesting a role for TUBA4A in the huoMTOC formation (Fig. 2I). Interestingly, the *TUBA4A* mutant did not affect mouse preimplantation development as severely as in human embryos, indicating that TUBA4A may have different roles in mouse aMTOCs and huoMTOCs, or the mitosis checkpoint of human zygotes might be more stringent than that of mice (Fig. 2I).

Among the three cases of TUBA4A mutation, E284G has not been reported before, while E284K was also listed in a recent paper by Li et al., where they found that TUBA4A appears to be a *de novo* mutation in females causing infertility [8]. TUBA4A is specifically highly expressed in oocytes, sperms, and neuronal tissues, but all the heterozygous individuals are healthy without neurological disease based on informed consent health history surveys. Thus, TUBA4A E284G and E284K do not appear to affect most mitotic divisions of embryonic or adult cells or neural functions. The successful rescue of the TUBA4A mutant phenotype in mouse oocytes by transient overexpression of the WT protein might have potential therapeutic implications. All the heterozygous female carriers are infertile due to failure of meiosis or the first few mitotic divisions. Since the zygotes contain mostly maternal proteins inherited from the oocytes, WT and mutant proteins should be present in the carrier’s oocytes, the delivery of more WT TUBA4A protein through mRNA microinjection may be able to replace the mutant TUBA4A protein at the spindle and allow normal division.

In summary, our results suggest that human sperm with *de novo TUBA4A* mutations, if successfully fertilized in the egg, will lead to infertility of the female offspring. These findings have important implications for assisted human reproduction and genetic counseling of women with oocyte and zygote division failure.

## Research limitations

There are also some limitations to our study. In human female TUBA4A mutation carriers, the majority of retrieved oocytes reached metaphase II, while their zygotes failed to develop past the cleavage stage. On the contrary, in mouse oocytes, overexpression of both mutants significantly reduced the rate of successful meiosis I, but in zygotes, only slightly reduced the rate of blastocyte formation. These results suggest that there are species differences between humans and mice. Repeating similar experiments in other mammals would be interesting to determine whether TUBA4A mutants have similar effects. Moreover, the mechanism of how TUBA4A mutants disrupt proper spindle assembly needs further study.

## Supplementary Material

lnad032_suppl_Supplementary_Material
