# Supplementary material for: Mutations in TUBA4A lead to human zygotic arrest and early developmental failure
Source: Life Med. 2023 Aug 23;2(5):lnad032. doi: 10.1093/lifemedi/lnad032 (PMC11749853; doi:10.1093/lifemedi/lnad032)
Supplement: lnad032_suppl_Supplementary_Material [file lnad032_suppl_Supplementary_Material.pdf]

Mutations in *TUBA4A* lead to human zygotic arrest

and early developmental failure

Abbreviations

E284G	c.851G>A;p.E284G mutation of TUBA4A
E284K	c.850G>A;p.E284K mutation of TUBA4A
GV	Germinal vesicle
GVBD	Germinal vesicle breakdown
HGMD	Human Gene Mutation Database
IVF	*In vitro* fertilization
MI	Metaphase I
MII	Metaphase II
TUBA4A	Tubulin alpha 4a
TUBB8	Tubulin beta 8 class VIII
WT	Wild type

Supplemental Methods

Patients

The patients with zygotic arrest and female infertility were recruited from the First Affiliated Hospital of Anhui Medical University. The human sample collection and analysis were approved by the Ethics Committee for Clinical Medical Research of the First Affiliated Hospital of Anhui Medical University (No. PJ2020-13-10). Each patient signed the informed consent, and 5 mL of peripheral blood was collected.

Whole-exome sequencing (WES) analysis

WES was carried out as described (Chen, Guo et al. 2022). The process for variants filtering was as follows. Variants including missense, nonsense, frameshift, and splice-site with minor allele frequency < 1% in public databases, including Genome Aggregation Database, 1000 Genomes, and the NHLBI Exome Sequencing Project were retained. Then variants in genes

associated with oocyte maturation and early embryonic development or expressed highly during the GV, MI, MII, zygote, and 2-cell stage were mainly focused on.

Transcriptome and translatome analysis

Transcriptome analysis of TUBA4A in human and mouse GV oocyte to blastocyst used RNA-sequencing data from GSE36552, and GSE71434 (Yan, Yang et al. 2013, Zhang, Zheng et al. 2016). The translatome analysis was based on Ribo-lite data from GSE197265 and GSE165782 (Xiong, Xu et al. 2022, Zou, Zhang et al. 2022). Translational efficiency (TE) analysis was calculated by the ratio of Ribo-lite and mRNA-seq (FPKM+1/FPKM+1). TE > 3 was considered a high TE, and TE < 0.33 was considered a low TE.

Mouse oocyte collection, embryo culture, and microinjection

All animal experiments were conducted following the Guide for the Care and Use of Animals for Research Purposes. The protocol for mouse oocyte collection and embryo isolation was approved by the Institutional Animal Care and Use Committee and Internal Review Board of Tsinghua University. GV oocytes were collected from the ovary of 3 − 4 weeks old C57BL/6 females 48h after PMSG injection and then were incubated in M2 medium (Sigma M7167) supplemented with 10 μM Cilostamide (TargetMol 68550) at 37℃. Embryos were collected from wild-type C57BL/6 females (Charles River). Zygotes for mRNA microinjection were collected from mated female mice 20 h post-HCG, then cultured in KSOM medium (Merck Millipore MR-106-D) in a 37.5°C 5% CO_2 incubator. Microinjection of wild-type and mutant TUBA4A mRNAs at a concentration of 200 ng/μL into the mouse GV oocytes or zygotes was performed using Eppendorf FemtoJet and a Leica microscope micromanipulator. In the oocyte rescue experiment, WT and mutant TUBA4A mRNAs were co-injected at concentrations of 400 ng/μL and 200 ng/μL. In the zygote rescue experiment, WT and mutant TUBA4A mRNAs were co-injected at concentrations of 200 ng/μL and

200 ng/μL.

Plasmid cloning and RNA synthesis

Human and mouse *TUBA4A* cDNA was cloned into the RN3P vector for *in vitro* transcription of mRNA. mRNAs were generated using the T3 mMESSAGE mMACHINE Kit according to the manufactures instructions (Ambion).

Immunostaining and confocal microscopy

For oocyte immunostaining, mouse oocytes were fixed in 1% PFA at 4℃ overnight, then permeabilized with 0.25% Triton X-100 for 20 min. Embryos were then blocked with 5% BSA at room temperature for 2 h and incubated with primary antibody overnight at 4℃, followed by washing in PBST and incubating with secondary antibody for 1 h at room temperature. DNA was stained by DAPI (Sigma-Aldrich, 28718-90-3). For sperm immunostaining, selected spermatozoa were washed with phosphate-buffered saline (PBS) and were fixed with 4% paraformaldehyde for 30 min at room temperature. Then, fixed spermatozoa were smeared on slides pre-dripped with poly-lysine, followed by permeabilization with 0.5% Triton X-100 for 15 min and blocking with 5% BSA for 1 h at room temperature. Then the slides were incubated with primary antibody overnight at 4℃. The slides were sequentially washed with PBS and incubated with a secondary antibody for 1 h at room temperature. Finally, the slides were counterstained with DAPI to label DNA for 10 min at room temperature. Antibodies used in this study are: TUBA4A (Abcam, ab228701), a-tubulin (SCBT, sc53029). Secondary antibodies are Dylight 549-Goat Anti-Rat (Earthox, E032340) and Dylight 488-Goat Anti-Rabbit (Earthox, E032220). Immunostaining images were acquired with a Nikon A1R HD25 confocal microscope using an oil immersion 60× objective. Raw data were processed using open-source image analysis software Fiji ImageJ.

Ethics approval

The human sample collection and analysis were approved by the Ethics Committee for Clinical Medical Research of the First Affiliated Hospital of Anhui Medical University (No. PJ2020-13-10). All animal experiments were conducted following the Guide for the Care and Use of Animals for Research Purposes. The protocol for mouse embryo isolation was approved by the Institutional Animal Care and Use Committee and Internal Review Board of Tsinghua University.

Statistical analysis

Data are presented as mean ± standard error of the mean (SEM). Statistical significance was determined by Student's *t*-test (two-tail) for two groups; one-way or two-way Analysis of Variance (ANOVA) for multiple groups using Graphpad prism8 software. $P < 0.05$ was considered significant.

Data availability

All data generated or analyzed during this study are included in this published article (and its supplementary information files).

Consent to participate

The human sample collection and analysis were approved by the Ethics Committee for Clinical Medical Research of the First Affiliated Hospital of Anhui Medical University and carried out with informed consent.

Consent for publication

The participant has consented to the submission of the study to the journal.

References

Chen, B., J. Guo, T. Wang, Q. Lee, J. Ming, F. Ding, H. Li, Z. Zhang, L. Li, Y. Cao and J. Na (2022). "Maternal heterozygous mutation in CHEK1 leads to mitotic arrest in human zygotes." Protein Cell **13**(2): 148-154.

Xiong, Z., K. Xu, Z. Lin, F. Kong, Q. Wang, Y. Quan, Q. Q. Sha, F. Li, Z. Zou, L. Liu, S. Ji, Y. Chen,
H. Zhang, J. Fang, G. Yu, B. Liu, L. Wang, H. Wang, H. Deng, X. Yang, H. Y. Fan, L. Li and W. Xie (2022). "Ultrasensitive Ribo-seq reveals translational landscapes during mammalian oocyte- to-embryo transition and pre-implantation development." Nat Cell Biol **24**(6): 968-980.

Yan, L., M. Yang, H. Guo, L. Yang, J. Wu, R. Li, P. Liu, Y. Lian, X. Zheng, J. Yan, J. Huang, M. Li,
I. Wu, L. Wen, K. Lao, R. Li, J. Qiao and F. Tang (2013). "Single-cell RNA-Seq profiling of human preimplantation embryos and embryonic stem cells." Nat Struct Mol Biol **20**(9): 1131-1139.

Zhang, B., H. Zheng, B. Huang, W. Li, Y. Xiang, X. Peng, J. Ming, X. Wu, Y. Zhang, Q. Xu, W.
Liu, X. Kou, Y. Zhao, W. He, C. Li, B. Chen, Y. Li, Q. Wang, J. Ma, Q. Yin, K. Kee, A. Meng, S. Gao, F. Xu, J. Na and W. Xie (2016). "Allelic reprogramming of the histone modification H3K4me3 in early mammalian development." Nature **537**(7621): 553-557.

Zou, Z., C. Zhang, Q. Wang, Z. Hou, Z. Xiong, F. Kong, Q. Wang, J. Song, B. Liu, B. Liu, L. Wang,
F. Lai, Q. Fan, W. Tao, S. Zhao, X. Ma, M. Li, K. Wu, H. Zhao, Z. J. Chen and W. Xie (2022). "Translatome and transcriptome co-profiling reveals a role of TPRXs in human zygotic genome activation." Science **378**(6615): abo7923.

Supplemental Figure Legends

Figure S1. TUBA4A is conserved among mammals.

(A) Genome locus of human *TUBA4A* and conserved sequences among different species.

(B) The amino acid sequence around TUBA4A E284 among different mammals.

(C and D) Translation efficiency of TUBA4A in human (C) and mouse (D) oocytes.

(E and F) Immunostaining of TUBA4A in mouse (E) and human (F) sperms. Cyan, DAPI; yellow, α-tubulin; magenta, TUBA4A. Scale bar, 10 m.

Figure S2. Overexpression of TUBA4A E284K and E284G mutants leads to reduced 1st PB protrusion in mouse oocytes.

(A and B) Bright-field and GFP images of oocytes injected with TUBA4A WT, E284K, and E284G mutants (as indicated). Images were taken 4 or 20 h after injection. Oocytes with the 1st polar body were marked with a red *.

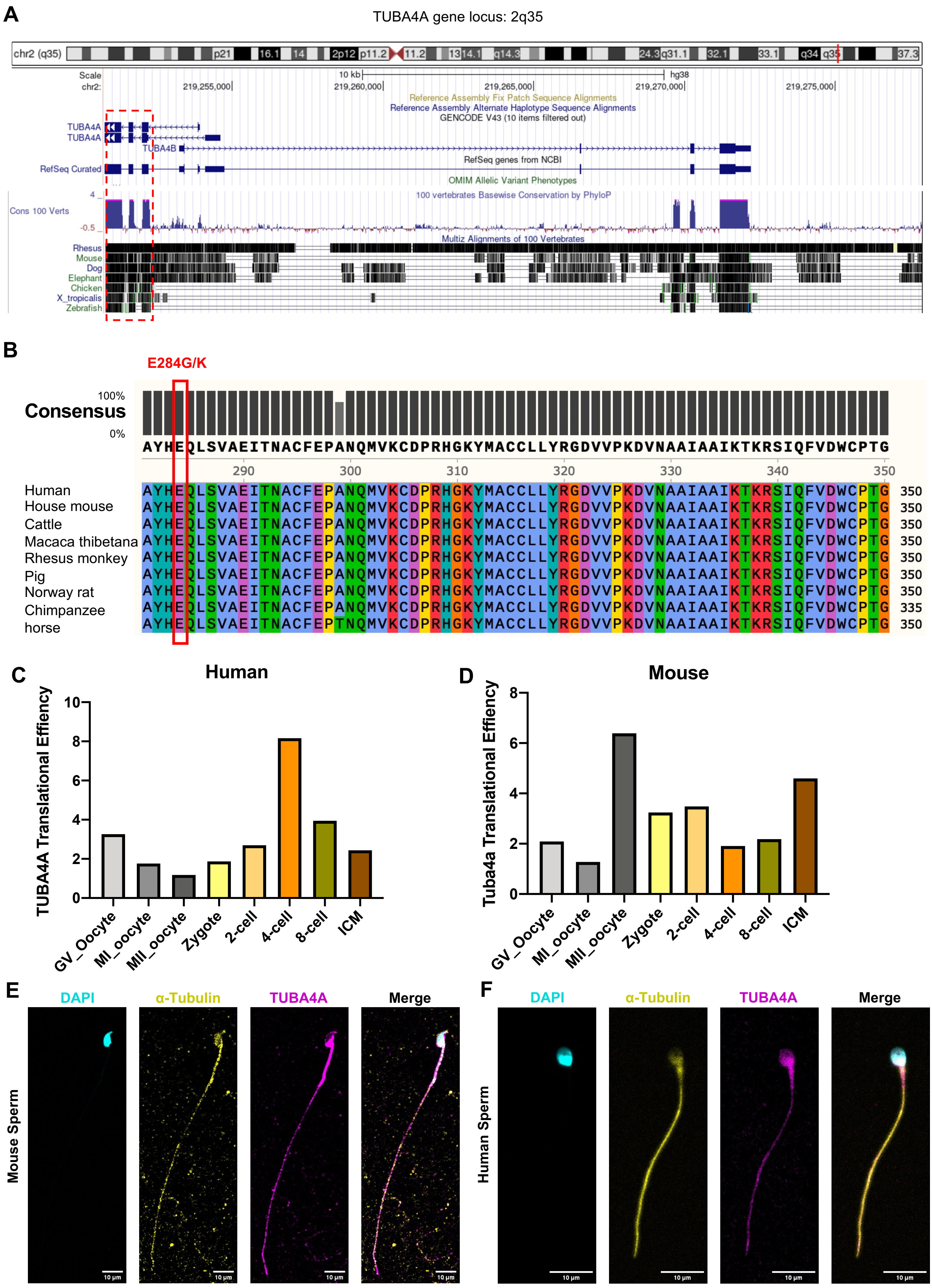
A
TUBA4A gene locus: 2q35
chr2 (q35)
Scale
chr2:
10 kb
hg38
219,255,000
219,260,000
219,265,000
219,270,000
219,275,000
Reference Assembly Fix Patch Sequence Alignments
Reference Assembly Alternate Haplotype Sequence Alignments
GENCODE V43 (10 items filtered out)
TUBA4A
TUBA4A
TUBA4B
RefSeq genes from NCBI
RefSeq Curated
OMIM Allelic Variant Phenotypes
100 vertebrates Basewise Conservation by PhyloP
Cons 100 Verts
4
-0.5
Multiz Alignments of 100 Vertebrates
Rhesus
Mouse
Dog
Elephant
Chicken
X_tropicalis
Zebrafish
B
E284G/K
100%
Consensus
0%
AYHEQLSVAEITNACFEPANQMVKCDPRHGKYMACCLLYRGDVVPKDVNAAIAAIKTKRSIQFVDWCPTG
290
300
310
320
330
340
350
Human AYHEQLSVAEITNACFEPANQMVKCDPRHGKYMACCLLYRGDVVPKDVNAAIAAIKTKRSIQFVDWCPTG 350
House mouse AYHEQLSVAEITNACFEPANQMVKCDPRHGKYMACCLLYRGDVVPKDVNAAIAAIKTKRSIQFVDWCPTG 350
Cattle AYHEQLSVAEITNACFEPANQMVKCDPRHGKYMACCLLYRGDVVPKDVNAAIAAIKTKRSIQFVDWCPTG 350
Macaca thibetana AYHEQLSVAEITNACFEPANQMVKCDPRHGKYMACCLLYRGDVVPKDVNAAIAAIKTKRSIQFVDWCPTG 350
Rhesus monkey AYHEQLSVAEITNACFEPANQMVKCDPRHGKYMACCLLYRGDVVPKDVNAAIAAIKTKRSIQFVDWCPTG 350
Pig AYHEQLSVAEITNACFEPANQMVKCDPRHGKYMACCLLYRGDVVPKDVNAAIAAIKTKRSIQFVDWCPTG 350
Norway rat AYHEQLSVAEITNACFEPANQMVKCDPRHGKYMACCLLYRGDVVPKDVNAAIAAIKTKRSIQFVDWCPTG 350
Chimpanzee AYHEQLSVAEITNACFEPANQMVKCDPRHGKYMACCLLYRGDVVPKDVNAAIAAIKTKRSIQFVDWCPTG 335
horse AYHEQLSVAEITNACFEPTNQMVKCDPRHGKYMACCLLYRGDVVPKDVNAAIAAIKTKRSIQFVDWCPTG 350
C
Human
TUBA4A Translational Effiency
0
2
4
6
8
10
GV_Oocyte
MI_oocyte
MII_oocyte
Zygote
2-cell
4-cell
8-cell
ICM
D
Mouse
Tuba4a Translational Effiency
0
2
4
6
8
GV_Oocyte
MI_oocyte
MII_oocyte
Zygote
2-cell
4-cell
8-cell
ICM
E
DAPI
α-Tubulin
TUBA4A
Merge
Mouse Sperm
10 μm
F
DAPI
α-Tubulin
TUBA4A
Merge
Human Sperm
10 μm

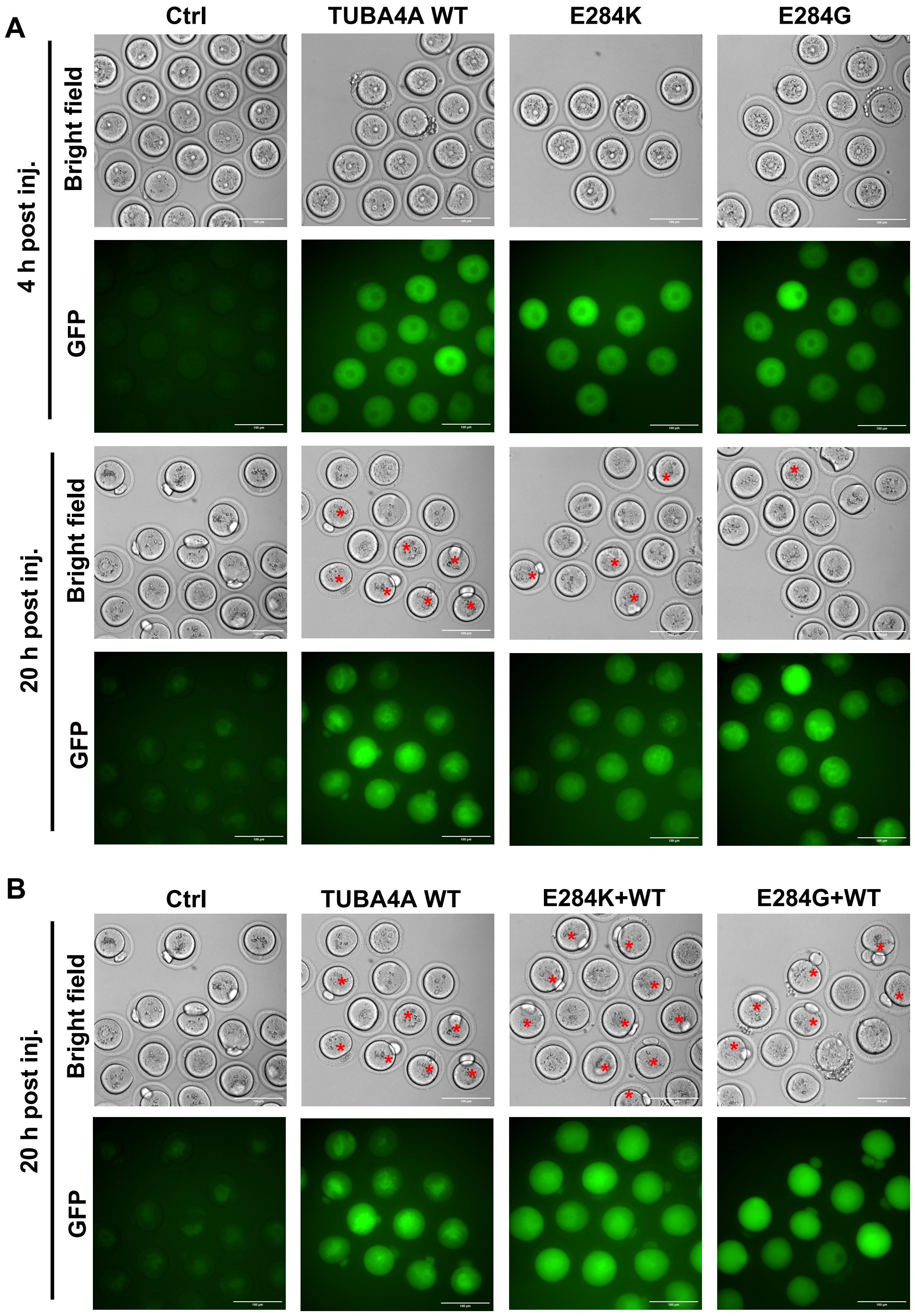

A
Ctrl
TUBA4A WT
E284K
E284G
4 h post inj.
Bright field
GFP
20 h post inj.
Bright field
GFP
B
Ctrl
TUBA4A WT
E284K+WT
E284G+WT
20 h post inj.
Bright field
GFP
